# Supplementary material for: Active site specificity profiling datasets of matrix metalloproteinases (MMPs) 1, 2, 3, 7, 8, 9, 12, 13 and 14
Source: Data Brief. 2016 Feb 22;7:299–310. doi: 10.1016/j.dib.2016.02.036 (PMC4777984; doi:10.1016/j.dib.2016.02.036)
Supplement: Supplementary file 10 — Supplementary material [file mmc10.zip › WebPICS_hMMP14_G_1%/P3.html]

 

PICS results


|  |  |
| --- | --- |
| **P3\_A**  34 in 213 sites   16.0 %    effects > 10 perc. pnts.  (vice-versa in brackets)  P2\_A: -11.7 (-12.8)   P2prime\_L: 15.3 (17.3) |  |
| **P3\_C**  6 in 213 sites   2.8 %    effects > 10 perc. pnts.  (vice-versa in brackets)  P1\_H: 28.1 (15.4)   P1prime\_C: 29.1 (19.4)   P3prime\_M: 13.4 (11.5)   P3prime\_Q: 44.4 (22.2) |  |
| **P3\_H**  5 in 213 sites   2.3 %    effects > 10 perc. pnts.  (vice-versa in brackets)  P1prime\_M: 33.0 (11.0)   P2prime\_F: 33.9 (13.1) |  |
| **P3\_I**  16 in 213 sites   7.5 %    effects > 10 perc. pnts.  (vice-versa in brackets)  P1\_N: 36.9 (21.1)   P2prime\_N: 26.5 (42.5) |  |
| **P3\_P**  27 in 213 sites   12.7 %    effects > 10 perc. pnts.  (vice-versa in brackets)  P1\_A: 17.8 (14.6)   P1prime\_V: 11.5 (20.6)   P2\_K: 16.5 (22.3) |  |
| **P3\_V**  27 in 213 sites   12.7 %    effects > 10 perc. pnts.  (vice-versa in brackets)  P2\_S: 12.8 (17.3)   P2prime\_K: 23.7 (12.3)   P3prime\_K: 18.3 (20.6) |  |
